# Therapeutic landscape of Fabry disease: advances and challenges from classical strategies to emerging therapies

**DOI:** 10.3389/fmed.2025.1662867

**Published:** 2025-10-31

**Authors:** Miao Zhang, Chendan Wang

**Affiliations:** ^1^The Fifth Clinical Medical College of Shanxi Medical University, Taiyuan, China; ^2^Shanxi Provincial Key Laboratory of Kidney Disease, Taiyuan, China; ^3^Medicinal Basic Research Innovation Center of Chronic Kidney Disease, Ministry of Education, Shanxi Medical University, Taiyuan, China

**Keywords:** Fabry disease, enzyme replacement therapy, pharmacological chaperone therapy, substrate reduction therapy, mRNA therapy, AAV-mediated gene therapy

## Abstract

Fabry disease (FD), as an X-linked lysosomal storage disorder (LSD), has seen significantly improved in patient prognosis since enzyme replacement therapy (ERT) was applied clinically in 2001. However, ERT has drawbacks such as immunogenicity, individual efficacy variability, and long-term treatment burdens. With deeper insights into the multidimensional pathological mechanisms of FD and breakthroughs in new delivery systems (such as mRNA therapy, engineered adeno-associated virus vectors), various emerging therapies have gradually developed. Nevertheless, each therapeutic strategy still faces areas needing improvement due to high disease heterogeneity, cytotoxicity, insufficient targeted tissue delivery efficiency, and a lack of long-term safety data. This article systematically reviews the development of different treatment strategies for FD, outlines the evolution from ERT clinical application to emerging technologies like novel vector delivery, analyzes the technical breakthroughs and clinical limitations of therapies at each stage, reveals the intrinsic connection between deepened understanding of pathological mechanisms and innovative treatments, and explores potential optimization directions for future treatment strategies based on global accessibility data.

## Graphical abstract

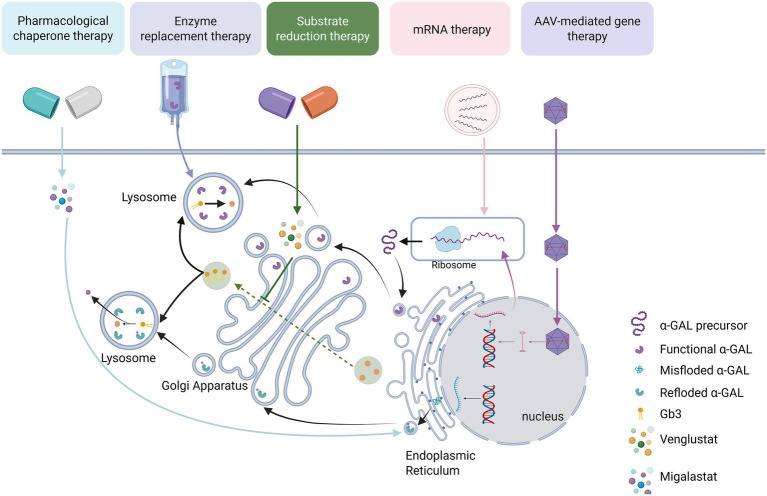

## 1 Introduction

Fabry disease (FD) is a rare X-linked lysosomal storage disorder (LSD) with an estimated birth prevalence of 1 in 6,264 based on a large-scale Chinese newborn-screening cohort (1). FD is caused by pathogenic variants in the *GLA* gene that reduce or absent the activity of the encoded lysosomal enzyme *α*-galactosidase A (α-Gal A). Consequently, the metabolic substrates globotriaosylceramide (Gb3) and its deacylated derivative globotriaosylsphingosine (lyso-Gb3) accumulate in multiple cell types—including endothelial cells, podocytes, smooth-muscle cells, cardiomyocytes, and neurons (2, 3). Progressive accumulation underlies the multi-systemic pathology of FD. *GLA* variants encompass missense, nonsense, frameshift, splice-site, and large copy-number alterations. Missense mutations are the most common, and over 1,000 different genotypes have been identified (4). FD can be classified into classic and non-classic/late-onset forms based on clinical presentation, with a clear distinction between the two types in males. The classic form is typically characterized by absent or significantly decreased *α*-Gal A enzyme activity, with symptoms usually beginning in childhood or adolescence and progressing to renal, cardiac, and central nervous system pathology in adulthood. In contrast, late-onset patients, retaining higher residual enzyme activity, experience milder phenotypes and a delayed disease course (Table 1). Plasma Lyso-Gb3 levels help to differentiate between the classic and late-onset forms of FD, with classic patients typically exhibiting significantly higher Lyso-Gb3 levels than late-onset patients (5). Due to the stochastic and dynamic nature of X-chromosome inactivation (XCI), and mechanisms such as skewed XCI, female patients exhibit marked heterogeneity in disease severity, ranging from asymptomatic to severe classic manifestations (6). Nevertheless, XCI pattern alone fails to predict individual phenotype. In a single-centre, cross-tissue study of 95 female *GLA*-variant carriers, skewed XCI (≥75:25) was detected in 35% of blood, 33% of skin fibroblast and only 7% of oral epithelial samples. Irrespective of tissue source or XCI pattern (skewed vs. random, mutant-allele-active vs. inactive), no significant association emerged between XCI skewing and clinical severity, organ involvement, leukocyte *α*-Gal A activity, or plasma Gb3 (*p* > 0.05). Thus, even in blood—the tissue most prone to skewing—XCI status does not stratify symptom severity (7).

**Table 1:** Fabry disease genotype–phenotype spectrum and clinical characteristics overview.

Phenotypes	Mutation type		Representative genotypes	PMID	Plasma α-GAL activity	Organotypic phenotypes focus	Initial symptoms/Core clinical manifestations
Classic Fabry disease	Exon mutation	Severe missense mutation	p.R112C, p.A156V, p.L166V, p.G260A, p.G373S, p.D266E, p.A37V	30386727, 7575533, 12428061, 20300124	Significantly reduced (far below normal levels)	Multi-organ involvement, including heart, kidney, brain, skin, eyes, etc	Peripheral sensory abnormalities, cutaneous angiokeratomas, corneal vortex opacity, hypohidrosis appear in childhood or adolescence; left ventricular hypertrophy, chronic kidney disease, and stroke develop as the disease progresses
		Missense mutation	p.R220*, p.R227*, p.Q386*, p.W262*, p.R301*, p.C378*, p.E7*, p.Q283*, p.W236*, p.W245*, p.W162*, p.Q250*, p.R301*, p.Y173*, p.W44*, p.W81*, p.E203*, p.W204*, p.W236*, p.W245*	38002959, 12428061, 10649504, 7951217, 7504405, 12428061, 23307880			
		Exon fragment deletion	58del15, exon1 deletion, exon7 deletion	16595074, 22731890, 31020209, 32612933			
		Frame-shift mutation	c.296del2, c.802del4, c.448delG	11076046, 33732617			
	Site-directed mutation		IVS4-1G>A, IVS5-2A>G, IVS5+3A>G, IVS5+4A>G, IVS6-1G>C, IVS5+1G>T	16595074, 12938095			
	Deep intronic mutation		IVS4+1326C>T, c.640-814T>C	35725559, 39613053			
Late-onset fabry disease	Missense mutation		p.R112H, p.N215S, p.A143T, p.R356W, p.Phe113Leu, GLA-Thr410Ala, p.D313Y, p.G195V, p.M296I, p.F113L, p.Q279E, p.G144D	34803097, 40898253, 40692291, 37619881, 37097439, 36927868, 36344272, 34067605, 32099817, 21972175, 28224042	Mild reduction (partial activity retained)	Predominantly involves a single or a few organs, commonly the heart and kidneys	Onset in adulthood, initial symptoms are mostly left ventricular hypertrophy, proteinuria (progressing to chronic kidney disease), and less commonly the classic sensory abnormalities of the skin, eyes, or extremities
	Deep intronic mutation		IVS4 + 919G > A	39308653			
Functional polymorphism	Missense mutation		p.E66Q, c.-10C>T	40823509, 30386727, 30201457, 28275245, 27160240, 26179544, 24718812	Normal or mild reduction	No clear organ-specific involvement, or only associated with specific mild phenotypes	No classic clinical manifestations of FD; some studies suggest it may be associated with an increased risk of small vessel brain occlusion in elderly males, but there are no characteristic symptoms of lysosomal lipid deposition related to FD; kidney biopsy shows no characteristic pathological changes of FD

The disease is characterized by insidious early symptoms and multisystem involvement, making early diagnosis challenging. The median delay from first symptom to confirmed diagnosis is 15 years (8). Renal biopsies from nine paediatric patients with only trace proteinuria already showed widespread Gb3 deposition, independent of overt nephropathy. Structural changes in the kidneys, including glomerulosclerosis and interstitial fibrosis, were observed in nearly 80% of patients. These findings suggest that severe pathological damage to the target organs may already be present early in the disease course (9). Patients who initiated enzyme replacement therapy (ERT) in childhood or early adulthood exhibited a slower progression of renal and cardiac disease compared to those who began ERT in late adulthood (10, 11). The introduction of dried-blood-spot (DBS) screening has now streamlined newborn and high-risk detection, enabling a genuine “early-diagnosis, early-treatment” paradigm.

Gb3 accumulates in glomerular endothelial, mesangial and podocyte compartments as well as peritubular capillaries (12). This accumulation can cause cellular dysfunction, which in turn leads to impaired renal filtration. As the disease progresses, the kidneys can develop fibrosis and scar formation, eventually leading to end-stage renal disease (13). Proteinuria is a common manifestation of renal involvement in FD, often presenting as microproteinuria in the early stages. The degree of proteinuria increases progressively with disease advancement. Massive proteinuria is not only a marker of renal injury but also exacerbates the renal burden, accelerating the deterioration of renal function (14, 15).

Gb3 and its metabolites accumulate extensively in cardiac tissues, interfering with normal cellular metabolic processes. This accumulation leads to dysfunction and injury of cardiomyocytes and cardiac vascular endothelial cells (16). Damage to the endothelial cells of cardiac blood vessels results in vessel wall thickening and luminal narrowing, thereby affecting myocardial blood supply (17). Left ventricular hypertrophy (LVH), characterized by increased left ventricular wall thickness, is a common manifestation of cardiac involvement in FD (18). This thickening can impair both diastolic and systolic cardiac function. As the disease progresses and the heart undergoes chronic damage, myocardial contractile and diastolic functions are progressively compromised, potentially leading to heart failure (19).

In the nervous system, patients with FD experience a variety of neurologic symptoms, including neuropathic pain, impaired sweating, and hearing deficit. Gb3 and its metabolites are primarily deposited in Schwann cells, dorsal root ganglia, and neurons within the central nervous system. This deposition interferes with the normal metabolic processes of nerve cells, leading to nerve fiber damage and disruption of nerve signaling (20). As the disease progresses, nerve cells may undergo pathological changes such as degeneration and apoptosis, leading to neurological dysfunction. Pain, a common symptom of neurological involvement, is typically manifested as episodes of severe pain, including tingling, burning, or electric shock-like sensations, often localized to the extremities, particularly the hands and feet (21). Additionally, the accumulation of Gb3 triggers inflammatory responses and oxidative stress, which further exacerbate target organ damage (22).

As research deepens and clinical experience accumulates, the clinical presentation spectrum of FD has been greatly expanded. As a disease affecting multiple systems, its manifestations are highly heterogeneous and camouflaged. More and more atypical or easily overlooked symptoms, such as decreased lung function (23), gastrointestinal manifestations (abdominal pain, diarrhea, vomiting) (24, 25), and azoospermia (26, 27), have been identified, sometimes even as the initial or only symptom, greatly challenging traditional diagnostic approaches. Since ERT was introduced into clinical treatment, emerging therapies that are continuously iterated are gradually reshaping its treatment landscape. From the initial ERT “alternative therapy” aimed at supplementing exogenous *α*-Gal A, to the “functional repair therapies” represented by small-molecule pharmacological chaperones and substrate reduction therapy, and then to the “root cause correction therapies” centered on gene therapy, mRNA delivery, and gene editing, the treatment philosophy has undergone three generational leaps. Registered clinical studies regarding the emerging therapies of FD are summarized in Table 2.

**Table 2:** Registered clinical studies regarding the emerging therapies of FD.

ClinicalTrials.gov, identifier	Study title	Study phase	Actual/estimated enrollment	Primary outcome	Location	Status
NCT05710692	Study to evaluate the safety, PK, PD, and efficacy of PRX-102 in Japanese patients with Fabry disease (RISE)	Phase 2/3	18	Incidence of Treatment Emergent Adverse Events (TEAEs)	Japan	Recruiting
NCT06328608	A study to learn about the safety and effects of the study drug PRX-102 in children and adolescents with Fabry disease (FLY)	Phase 2/3	22	Incidence of Treatment Emergent Adverse Events (TEAEs)	USA, Austria, France, Norway, Spain, UK	Recruiting
NCT03180840	Safety, efficacy, and PK of PRX-102 in Patients with fabry disease administered intravenously every 4 Weeks (BRIGHT)	Phase 3	30	Treatment-emergent adverse events (TEAEs)	USA, Belgium, Czechia, Denmark, Italy, Norway, UK	Completed
NCT02930655	A study to assess the safety and tolerability of lucerastat in subjects with Fabry disease	Phase 1	14	Effecacy, safety and tolerability	Germany	Completed
NCT06114329	Study of the safety and biologic activity of AL01211 in Treatment Naive Males With classic Fabry disease	Phase 2	16	Treatment-emergent adverse events (TEAEs)	China	Recruiting
NCT02489344	Evaluation of the long-term safety, pharmacodynamics, and exploratory efficacy of GZ/SAR402671 in treatment-naïve adult male patients with Fabry disease	Phase 2	8	Treatment-emergent adverse events (TEAEs)	USA, France, Poland, Russia, UK	Completed
NCT03454893	Open Label, Study Of Efficacy and Safety Of AVR-RD-01 for treatment-naive subjects with classic Fabry disease	Phase 1/2	15	Adverse events (AEs) and serious adverse events (SAEs)	USA, Australia, Brazil	Terminated
NCT06207552	Evaluation of the safety, tolerability and efficacy of a gene therapy drug for the treatment of pediatric Fabry disease	Phase 1	6	Adverse events (AEs) and serious adverse events (SAEs)	China	Recruiting
NCT04046224	Dose-ranging study of ST-920, an AAV2/6 Human alpha galactosidase A gene therapy in subjects with Fabry disease (STAAR)	Phase 1/2	33	Treatment-emergent adverse events (TEAEs)	USA, Australia, Canada, Germany, Italy, UK	Completed
NCT06819514	The safety and efficacy of intravenous EXG110 in patients with Fabry disease	Phase 1/2	16	Effecacy, safety, tolerability, adverse events (AEs), serious adverse events (SAEs)	China	Not yet recruiting
NCT06270316	Safety, PK/PD, and exploratory efficacy study of AMT-191 in classic Fabry disease	Phase 1/2	12	Safety and tolerability, Incidence of Treatment-Emergent Adverse Events (TEAE)	USA	Recruiting
NCT05629559	4D-310 in adults with Fabry disease and cardiac involvement	Phase 1/2	18	Incidence and severity of adverse events	Australia	Recruiting

## 2 Enzyme replacement therapy

**Figure 1:**
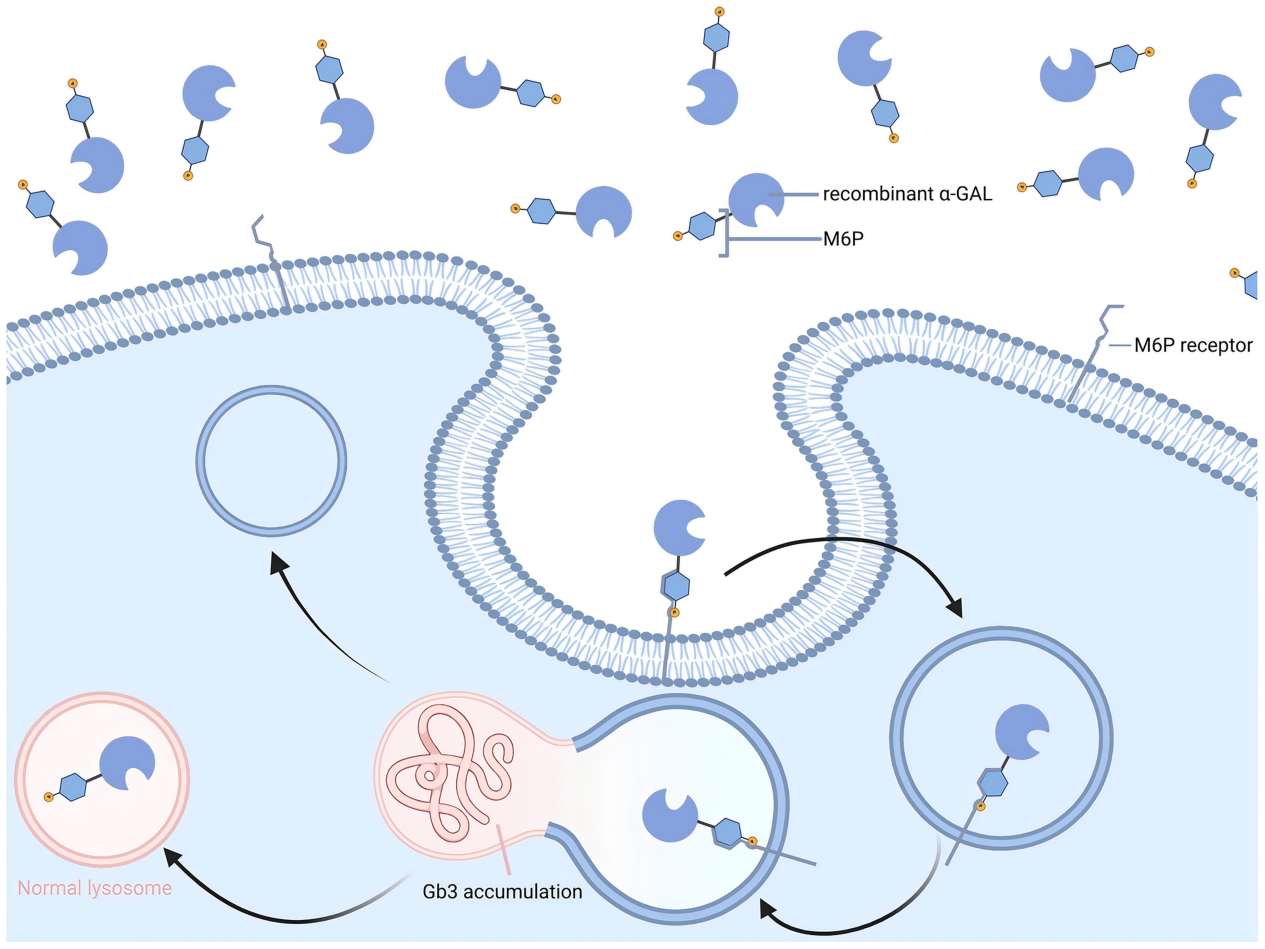
Enzyme replacement therapy for Fabry disease.

In 2001, ERT was introduced into clinical practice, based on the principle of promoting the degradation of Gb3 and Lyso-Gb3 through intravenous infusion of recombinant *α*-Gal A (Figure 1). Currently, three ERT drugs have been approved for clinical use worldwide: agalsidase alfa (Replagal®; Takeda), agalsidase beta (Fabrazyme®; Sanofi), and pegunigalsidase alfa (Elfabrio®; Chiesi) (28, 29).

In a 6-month randomized, double-blind, placebo-controlled trial, recombinant *α*-Gal A markedly reduced renal Gb3 deposition: mesangial widening fell by 12.5 ± 5.0% in the ERT group but rose by 16.5 ± 7.7% with placebo, yielding a 29% absolute difference (95% CI 8–50%, *p* = 0.01). Concurrently, mean creatinine clearance increased by 2.1 mL min^−1^ 1.73 m^−2^ with ERT and declined by 16.1 mL min^−1^ 1.73 m^−2^ with placebo, giving a between-group difference of 18.2 mL min^−1^ 1.73 m^−2^ (95% CI 2.6–33.8, *p* = 0.02) (30). In a 20-year observational study based on Fabry Outcome Survey (FOS), adult patients receiving ERT were indirectly compared with an untreated cohort. The results showed that the annual decline rate of eGFR in the treatment group was significantly lower than that in the untreated group; among the eGFR subgroups: for males with baseline eGFR ≥60 mL min^−1^ 1.73 m^−2^, the annual decline rate in the treatment group was −1.68 ± 0.10 mL min^−1^ 1.73 m^−2^ yr.^−1^, and in the untreated group was −3.0 ± 0.1 mL min^−1^ 1.73 m^−2^ yr.^−1^ (*p* < 0.0001); for males with baseline eGFR <60 mL min^−1^ 1.73 m^−2^, the annual decline rate in the treatment group was −1.60 ± 0.34 mL min^−1^ 1.73 m^−2^ yr.^−1^, and in the untreated group was −6.8 ± 1.5 mL min^−1^ 1.73 m^−2^ yr.^−1^; stratified by baseline proteinuria, regardless of the patient’s 24-h urine protein concentration being ≥1.0 g, 0.1- < 1.0 g, or <0.1 g, the annual decline rate of eGFR in the treatment group was significantly lower than that in the corresponding untreated subgroup. Therefore, ERT treatment can significantly slow down the progression of renal function deterioration in FD patients; even when further subdivided by treatment duration, the decline rate of eGFR in patients with more than 10 years of ERT still shows an advantage, and the overall decline rate in the treatment group has always been lower than that in the untreated cohort. Among adult treated patients, those with treatment duration less than 10 years had an annual decline rate of eGFR of −1.42 ± 0.06 mL min^−1^ 1.73 m^−2^ yr.^−1^, and those with treatment duration over 10 years had an annual decline rate of −1.10 ± 0.08 mL min^−1^ 1.73 m^−2^ yr.^−1^, both significantly lower than the corresponding levels in the untreated group (31). In a retrospective analysis of 21 years of clinical data for male patients receiving agalsidase alfa 0.2 mg/kg intravenous infusion every other week in the FOS cohort, those with baseline proteinuria ≤0.5 g/24 h (*n* = 114) had an average annual eGFR loss of −1.61 mL min^−1^ 1.73 m^−2^; those with proteinuria >0.5 g/24 h (*n* = 51) had an average annual decline of −3.62 mL min^−1^ 1.73 m^−2^, with a difference of 2.01 mL min^−1^ 1.73 m^−2^ yr.^−1^ (95% CI 1.20–2.82, *p* < 0.0001), suggesting that early initiation of ERT in the early stage of renal damage can significantly slow down subsequent renal function decline (32).

A retrospective FOS analysis (NCT03289065) enrolled 560 males on agalsidase alfa who started ERT at ≤18 years (*n* = 151); baseline LVMI averaged 36.7 ± 7.9 g/m^2^ and only 6.8% exceeded the 50 g/m^2^ pathological threshold. Median follow-up was 6.3 ± 4.3 years. Long-term follow-up results showed that the annual change slope of LVMI in Cohort 1 was −0.17 g/m^2^ (95% CI: −0.66 ~ 0.31), although the statistical test *p*-value was 0.490, the numerical trend and clinical significance indicated that LVMI not only did not increase but showed a continuous slight decrease, and the mean LVMI remained stable at 33.6 g/m^2^ (95% CI: 29.5 ~ 37.7) during the follow-up period, significantly lower than the baseline level (33). A decade-long FOS extension showed that baseline-non-LVH patients (females ≤48 g/m^2^, males ≤50 g/m^2^) exhibited minimal LVMI increases: +0.52 g/m^2^ yr.^−1^ (95% CI: −0.13 ~ +1.17) and +0.57 g/m^2^ yr.^−1^ (95% CI: +0.02 ~ +1.13) for females and males, respectively, indicating absence of significant progression. At the same time, the annual change slope of posterior wall thickness at end-diastole (PWTd) in different baseline renal function subgroups was controlled at +0.01 ~ +0.11 mm yr.^−1^, remaining within the normal range, further confirming the long-term stable effect of ERT on cardiac structure (34). Nevertheless, sudden cardiac death and other major adverse cardiac events (MACE) continue to occur on ERT (35), indicating that decelerated progression does not equate to full arrhythmic or microvascular risk elimination.

ERT can effectively alleviate pain symptoms in FD patients and improve their autonomic function to some extent. Systematic reviews of relevant studies have demonstrated significant improvement in neuropathic pain and benefit for stroke prevention among patients treated with ERT (28, 36, 37). Several clinical trials have well documented significant reductions in pain scores, as well as in the frequency and severity of pain episodes, in patients treated with agalsidase alfa. ERT treatment increases the threshold for detecting hot and cold sensations in the hands and feet and improves sweating and heat tolerance (38, 39). Despite these positive results, ERT does not fully normalize the function of the peripheral nervous system (20).

The majority of patients currently follow a conventional dosing regimen. However, available studies continue to yield divergent conclusions regarding the correlation between dose and efficacy. A 53-week, III/IV phase, multicenter study (NCT01124643) indicates that in initially treated FD adults with left ventricular hypertrophy (LVMI >50 g/m^2^ for males, >47 g/m^2^ for females) at baseline, increasing the dosing frequency of agalsidase alfa from the approved 0.2 mg/kg every two weeks (EOW) to 0.2 mg/kg weekly, with LVMI as the primary endpoint—monitored at 53 weeks, EOW group +3.2 g/m^2^, weekly group +0.5 g/m^2^; LSM difference −2.20 g/m^2^ (95.005% CI –12.26 ~ 7.85, *p* = 0.6585), there was no statistically significant difference in changes between the two groups (40). However, for Fabry patients who have been regularly receiving 0.2 mg/kg agalsidase alfa infusion every two weeks and whose renal function continues to decline, switching to the same dose administered weekly significantly slowed the average decline rate of eGFR within 24 months; multivariate analysis shows that the weekly dosing frequency is the strongest independent predictor of improved eGFR slope (*β* = 0.6486, *p* = 0.0008) (41). A 14-year follow-up study of 20 classic FD patients found that the dose of ERT had a significant impact on efficacy. Although there was no significant difference in baseline renal function and proteinuria levels between the two groups before treatment, patients receiving higher doses showed more significant clearance of podocyte Gb3 deposits (average decrease of 23.16 points, *p* = 0.002), which was significantly higher than the low-dose group. In addition, the reduction of podocyte Gb3 was positively correlated with cumulative enzyme dose (*r* = 0.69, *p* = 0.001), and the high-dose group also showed a more optimal effect in the clearance of Gb3 in the intima of arteries/arterioles (*p* = 0.03). The residual plasma lysoGb3 levels were significantly lower in the high-dose group (10.4 vs. 20.1 nmol/L, *p* = 0.04) and were significantly associated with the cumulative dose in male patients (*r* = 0.71, *p* = 0.01) (42). Therefore, higher doses of agalsidase treatment can more effectively clear kidney Gb3 deposits, delay tissue damage, and support a clear dose-effect relationship between ERT dosing and the efficacy of FD. However, another 3-year follow-up observation systematically evaluated the efficacy changes after switching from agalsidase beta (1.0 mg/kg) to agalsidase alfa (0.2 mg/kg). The results showed that after the switch, patients’ cardiac structural indicators continued to significantly improve: the mean LVMI decreased from 58.1 g/m^2^ to 50.7 g/m^2^ (*p* = 0.045), while renal function remained stable, and plasma Gb3 and lyso-Gb3 levels did not significantly increase, and pain scores and quality of life also did not show significant changes. The above data indicate that even with a significant reduction in dose, efficacy was not weakened, and no evidence of additional efficacy improvement with increased dose was observed (43). The differences in the above conclusions mainly arise from the different observation time windows of the endpoint. Given that irreversible complications such as end-stage renal failure, stroke, and heart failure are concentrated at the end stage of the disease, existing switch or down-titration studies were mostly triggered by agalsidase-beta shortages and are limited to short-term surrogate endpoints. Consequently, “non-inferior” surrogate stability after dose reduction cannot yet be extrapolated to long-term hard-end-point risk. Therefore, current data are insufficient to inform long-term dosing strategies, and prospective, comparative trials with extended follow-up are urgently warranted.

Agalsidase alfa and agalsidase beta, the primary ERT agents for FD, exhibit differences in clinical efficacy and safety despite both being derived from recombinant *α*-Gal A. Sirrs et al. followed patients treated with agalsidase alfa and agalsidase beta for 10 years. The results showed that in male patients, the incidence of renal events (including renal replacement therapy, doubling of serum creatinine, and proteinuria >3.5 g/day) was lower in the agalsidase beta group than in the agalsidase alfa group. Additionally, the rate of decline in eGFR was slower in male patients treated with agalsidase beta (44). Krämer et al. conducted a study involving 112 patients with FD who received agalsidase beta for more than 1 year. In a subgroup analysis, patients were divided into two groups: the conversion group, which switched from agalsidase beta to agalsidase alfa, and the reconversion group, which switched back from agalsidase alfa to agalsidase beta. The results showed that the conversion group’s eGFR decreased by 4.6 ± 9.1 mL min^−1^ 1.73 m^−2^. In contrast, the reconversion group exhibited a significantly lesser decrease in eGFR after switching back to agalsidase beta. These findings suggest that agalsidase beta may be more advantageous than agalsidase alfa in maintaining stable renal function (45). In terms of safety, multiple studies suggest that the immunogenicity of *α*-galactosidase alfa is lower than that of α-galactosidase beta. Vedder et al. provided quantitative data from a prospective cohort study of 52 patients with FD: after 6 months of treatment, the positive rate of anti-α-Gal A antibodies in male patients increased with dose, with the α-galactosidase alfa 0.2 mg/kg group at 43% (4/10), the α-galactosidase beta 0.2 mg/kg group reaching 83% (6/8), and the α-galactosidase beta 1.0 mg/kg group even higher at 89% (8/10); overall comparisons showed that the antibody incidence in the beta 1.0 mg/kg group was significantly higher than that in the alfa 0.2 mg/kg group (*p* = 0.005). Further functional experiments showed that 83% of male patients’ serum could neutralize standard enzyme activity, and the antibody titer was highly correlated with ELISA results (*r* = 0.80, *p* < 0.001). Notably, no antibodies were detected in all female patients (*n* = 24), suggesting that gender is also an important covariate for immune response (46).

Pegunigalsidase alfa achieves molecular structure upgrade through dual modification of “chemical cross-linking-PEGylation”: Covalently bridging two molecules of *α*-Gal A with a 2000 Da branched PEG forms a homodimeric network, which has an 11 °C increase in thermal stability compared to the unmodified enzyme; in human plasma, the activity of the unmodified enzyme is almost completely lost after 30 min, while Pegunigalsidase alfa retains 30–40% activity after 30 min; under lysosome-like conditions, the activity of the unmodified enzyme remains only 10% after 10 days, while the modified Pegunigalsidase alfa maintains 80% activity, meeting the demand for administration every 2 weeks or even monthly; at the same time, the antibody binding curve slope of Pegunigalsidase alfa is smoother, proving that the PEG chain effectively shields the immunogenic epitopes, reduces antibody recognition, and lowers the risk of neutralizing antibody production. (47–49).

III phase cluster data (BALANCE, BRIGHT, BRIDGE, and 6-year extension) show that the 2-year eGFR slope is not worse than high-dose agalsidase beta (49); following switch from agalsidase alfa 0.2 mg/kg, annual eGFR slope improved from −5.9 to −1.19 mL min^−1^ 1.73 m^−2^ yr.^−1^ (50); plasma lyso-Gb3 maintained low levels, and renal capillary Gb3 inclusion bodies decreased by 84% (48); no new fibrosis occurred in the left ventricular mass index at 60 months (51). Across BALANCE, BRIDGE and real-world cohorts, exposure-adjusted infusion-reaction rates were 3.6- to 7.8-fold lower than with agalsidase beta; no de-novo neutralizing antibodies; 75% of the titers decreased in previous ADA-positive individuals after crossover; 97% of adverse events were mild to moderate, severe events <3%, and there were no deaths or III-IV grade hypersensitivity. In summary, pegunigalsidase alfa, with a chemical cross-linking-PEGylation strategy, simultaneously improves stability, extends half-life, and reduces immunogenicity (49–51). On the basis of efficacy non-inferior to high-dose beta preparations, it significantly improves safety and ease of administration, providing a better choice for ERT in FD.

## 3 Pharmacological chaperone therapy

**Figure 2:**
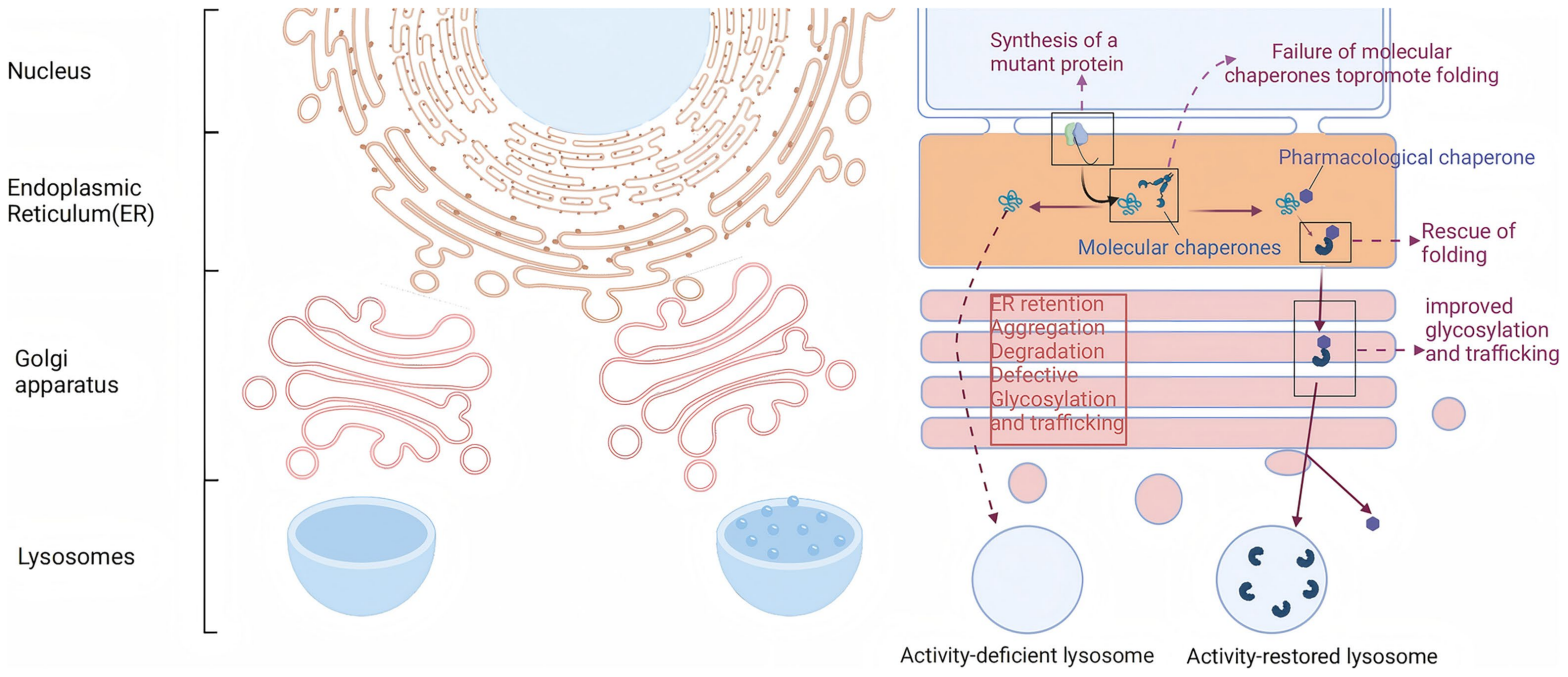
Migalastat stabilizes mutated *α*-Gal A enzymes to enhance enzymatic activity.

Approximately 35 to 50% of FD patients exhibit structural instability of the enzyme but retain some catalytic activity due to specific missense mutations in the *GLA* gene, such as p. N215S and p. F113L. Migalastat is a small molecule drug that is structurally similar to the substrate of *α*-Gal A, the Gb3-terminal galactose. Consequently, it can specifically and reversibly bind to the active site of α-Gal A mutants generated by *GLA* gene mutations, effectively providing an “auxiliary scaffold” for the mutated enzyme (Figure 2). This binding stabilizes the three-dimensional structure of the enzyme, preventing its recognition and degradation by the endoplasmic reticulum’s quality control system, thereby enhancing the stability of the mutant enzyme (52, 53). *In vitro* assays demonstrated that mutants were classified as therapeutic if they exhibited an absolute increase (≥ 3% of wild-type enzyme activity) and a relative increase (≥ 1.2-fold) in *α*-GAL activity after incubation with migalastat.

Long-term follow-up data show that for both naive and treated ERT patients, the oral migalastat 123 mg every other day regimen can significantly slow the decline in renal function. Summary of the FACETS, ATTRACT, and its open-label extension studies (median follow-up 5.9 years, longest 8.6 years, *n* = 78): after adjusting for gender, age, and baseline renal function with a random coefficients model, the mean annualized eGFR change in the naive ERT group was only −0.1 mL/min/1.73 m^2^, and in the treated ERT group was +0.1 mL/min/1.73 m^2^, both superior to the historical untreated cohort. Subgroup analysis showed that the mean annualized decline in classic male naive patients was −0.5 mL/min/1.73 m^2^, significantly lower than the −2.2 mL/min/1.73 m^2^ decline in the agalsidase beta 54-month cohort; those with baseline eGFR >90 mL/min had a decline of −0.2 mL/min/1.73 m^2^, and those with proteinuria <300 mg/24 h had a decline of −0.1 mL/min/1.73 m^2^ (54). Another paired renal biopsy study performed quantitative electron microscopy morphometry on 8 adult classic male patients carrying a potentially inducible *GLA* mutation, showing that after 6 months of oral migalastat 150 mg every other day treatment, the mean total Gb3 inclusion body volume in podocytes decreased from 4,200 ± 1,600 μm^3^ to 2,500 ± 1,100 μm^3^, with an average reduction of 41% (*p* = 0.02); at the same time, the mean podocyte volume decreased synchronously by 37% (*p* = 0.004), and the reduction in podocyte width was highly correlated with the decrease in Gb3 volume (*r* = 0.82, *p* = 0.02). Plasma lyso-Gb3 levels were also significantly associated with changes in podocyte Gb3 content (*r* = 0.79, *p* = 0.02) (55).

In a 18-month randomized, open-label, active-controlled phase III clinical trial (ATTRACT study, NCT00925301), a total of 57 adult patients with FD who had previously received ERT for ≥12 months were enrolled and randomly assigned in a 1.5:1 ratio to the oral drug migalastat group (*n* = 36, 150 mg once every other day) or to continue the original ERT regimen group (*n* = 21, agalsidase alfa 0.2 mg/kg or agalsidase beta 1.0 mg/kg, once every two weeks). After 18 months of treatment, the LVMI in the migalastat group significantly decreased from baseline by −6.6 g/m^2^ (95% CI −11.0 to −2.2, *p* < 0.01), while the ERT group showed only a non-significant change of −2.0 g/m^2^ (95% CI −11.0 to 7.0). Subgroup analysis showed that the migalastat recipients with pre-existing left ventricular hypertrophy (*n* = 13) had a more significant decrease, reaching −8.4 g/m^2^. This result was moderately correlated with the change in interventricular septum thickness (IVSWT) (r^2^ = 0.26, *p* = 0.0028) (56). In an 8.6-year observational study of migalastat treatment, it was found that although some patients still experienced cardiac-related events, overall cardiac function remained relatively stable (57). These findings suggest that migalastat has a protective and stabilizing effect on cardiac function by maintaining enzyme activity and reducing substrate buildup in the heart, potentially reducing the risk of heart disease progression.

Migalastat, the first oral agent for the treatment of FD, is now widely used in clinical practice. Compared to ERT, which requires frequent intravenous infusions, migalastat greatly improves patient convenience and compliance. However, the mechanism of action of migalastat limits its effectiveness to only some missense mutations (58). Recent studies have found that the *in vitro* treatability of mutations does not necessarily correspond to the *in vivo* treatability of migalastat-treated patients. This suggests that mutations shown to respond favorably to migalastat in in vitro assays may not have the desired effect in actual patient treatment (59). For example, in the national FD cohort in Switzerland, some patients carrying “combinable mutations” still had less effective treatment than ERT after receiving migalastat. A classic male patient (mutation p. S276N) switched from ERT to migalastat, and although the peripheral blood leukocyte *α*-Gal A activity increased from 3.0 to 9.9%, the plasma lyso-Gb3 level continued to rise, indicating insufficient substrate clearance, further deterioration of renal function, and ultimately, ERT was restarted due to “ineffective treatment” (58). Therefore, it is important to monitor patients’ clinical symptoms and laboratory markers during treatment to comprehensively assess the actual therapeutic efficacy of migalastat.

Migalastat is overall safe and well-tolerated in patients with FD. In the randomized controlled Phase III trial and its open-label extension study, patients treated with migalastat for up to 8.6 years did not show any new safety signals, serious adverse events were rare, and no patient discontinued due to drug-related toxicity (57). The Phase IIIb ASPIRE study in adolescents (*n* = 21, median age 14.7 years) also showed that migalastat’s tolerability was consistent with adults, and no unexpected adverse reactions specific to children were found (60). Additionally, the German multicenter MALTA-FABRY prospective cohort (*n* = 40, followed up for 24 months) reported that 92.5% of patients maintained high adherence, with only one case rated as low adherence due to gastrointestinal discomfort, and significant improvements in pain and physical function limitations (61). Notably, renal clearance removes approximately 74% of the dose during dialysis; pharmacokinetic study indicates that 123 mg every other day maintains therapeutic exposure in ESRD without adjustment or accumulation (62).

## 4 Substrate reduction therapy

**Figure 3:**
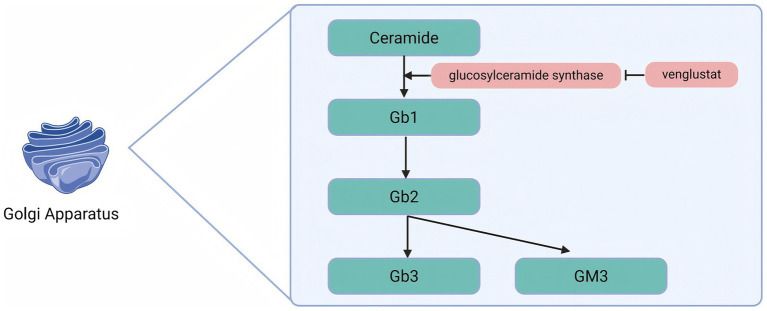
Venglustat inhibits glucose ceramide synthase to reduce Gb3 production.

Venglustat is an oral glucose ceramide synthase (GCS) inhibitor that acts by inhibiting the enzymatic conversion of ceramides to glucosylceramides, thereby reducing the available substrates for the synthesis of more complex glycosphingolipids (Figure 3).

Patients with FD suffer from *α*-Gal A deficiency, which leads to the progressive accumulation of Gb3 in the lysosomes of various cell types. By inhibiting GCS, Venglustat reduces the production of Gb1, which in turn decreases the synthesis of complex glycosphingolipids such as Gb3. This mechanism alleviates substrate accumulation at its source and mitigates the pathological process of FD. An international, open-label, single-arm, Phase 2a uncontrolled 26-week clinical trial (NCT02228460) and its 130-week extension study (NCT02489344) evaluated the safety, pharmacodynamics, pharmacokinetics, and exploratory efficacy of Venglustat in untreated adult male patients with classic FD. Patients received 15 mg of Venglustat orally once daily. Of the 11 patients enrolled, 9 completed the 26-week study, and 7 completed the extension study. At week 26, a decrease in Gb3 scores of the superficial skin capillary endothelium (SSCE) was observed in some patients. By the end of the extension study, Gb3 scores had decreased in 5 of 6 patients. The Gb3 inclusion body volume fraction in the cytoplasm of the SSCE, as measured by electron microscopy, was significantly lower at both week 26 and week 156 compared with baseline. In addition, Venglustat reduced the levels of markers associated with the major glycosphingolipid synthesis and degradation pathways, with a rapid decrease in proximal markers and a gradual decrease in distal markers. After 3 years of follow-up, no progression of FD was detected, confirming the target engagement and pharmacodynamic effects of Venglustat in this patient population (63).

As an oral drug, Venglustat offers the advantage of easy administration compared to traditional ERT, which can improve patient compliance. Due to its distinct pharmacological properties, Venglustat is able to cross the blood–brain barrier and reduce the levels of glycosphingolipids accumulated in the brain, an advantage that *α*-Gal A lacks (64). Therefore, the combination of Venglustat and ERT may provide complementary and additive therapeutic benefits.

## 5 mRNA therapy

**Figure 4:**
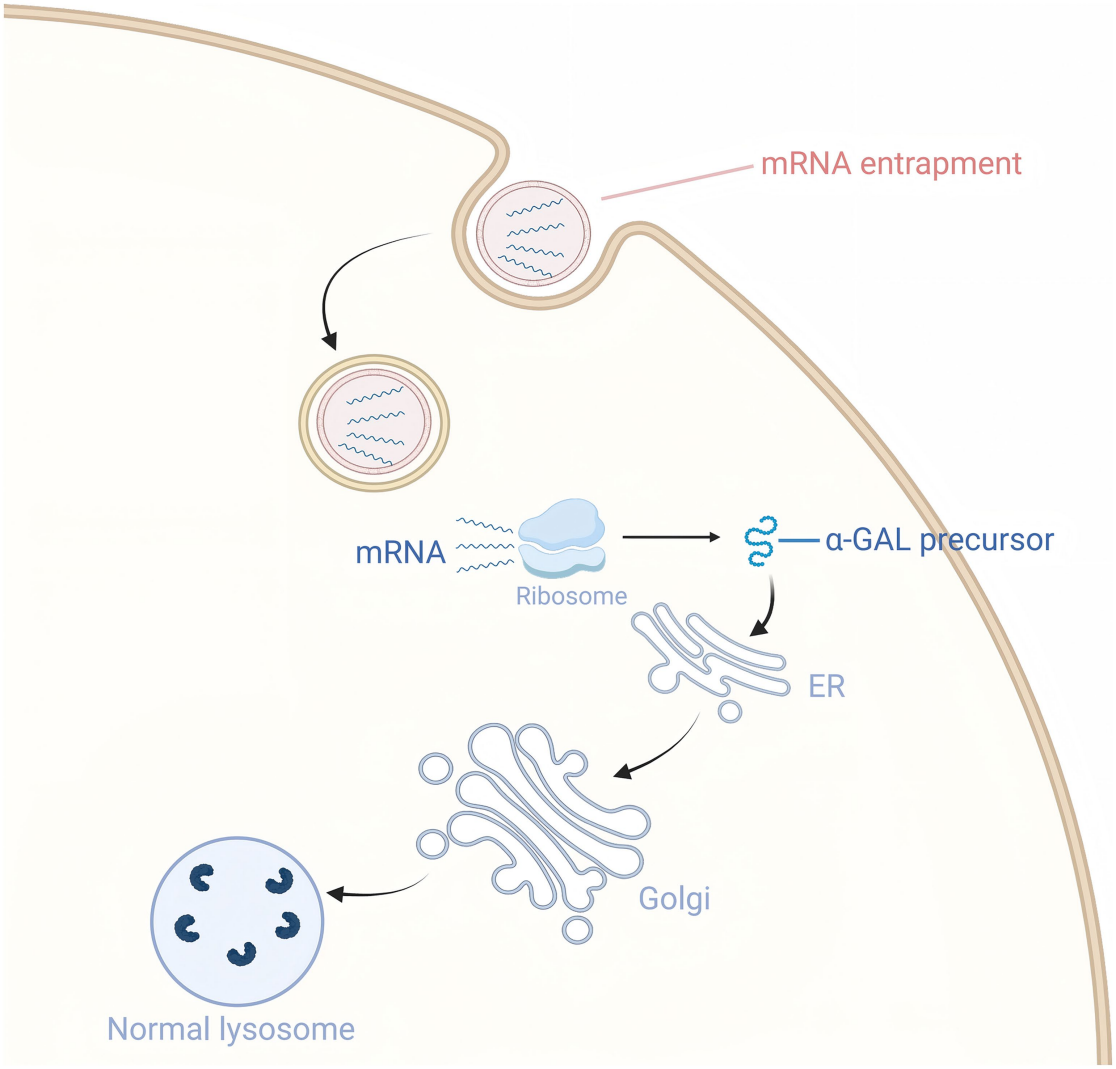
Exogenous mRNA delivery enhances α-GalA expression.

The application of mRNA therapy in FD treatment is primarily reflected in its ability to effectively increase α-Gal A levels and improve related symptoms (65, 66) (Figure 4). A preclinical study showed that intravenous injection of mRNA encoding human *α*-Gal A into wild-type mice, FD mouse models, and wild-type non-human primates resulted in significantly higher α-Gal A activity in the liver and heart of the 0.5 mg/kg dose group compared to wild-type mice after 72 h, with kidney activity reaching 50% of wild-type levels, and plasma activity being more than 500 times higher than wild-type after 6 h. It also reduced lyso-Gb3 levels in the liver and spleen by 80%, and in the kidneys and heart by more than 50%. After a single dose, the reduction effect on tissue substrates lasted for at least 6 weeks, and in the plasma for about 4 weeks. These results suggest that mRNA therapy is capable of sustained *in vivo* production of *α*-Gal A and effective reduction of substrate levels, indicating its potential applicability to the treatment of FD (67).

Based on evidence from multiple preclinical studies, repeated administration of LNP-encapsulated *α*-Gal A mRNA achieves sustained pharmacodynamic benefits and overall safety for Fabry disease. The Moderna team systematically evaluated the multi-dose regimen in *α*-Gal A knockout mice: the experimental group received intravenous injections of 0.2 mg/kg or 0.5 mg/kg *α*-Gal A mRNA every two weeks (EOW), or 0.5 mg/kg monthly, for 3 months; the control group was given an equivalent amount of eGFP mRNA. Organs were collected 7 days after the last dose, and liver IHC showed a dose-dependent increase in *α*-Gal A protein (0.5 mg/kg EOW > 0.5 mg/kg monthly > 0.2 mg/kg EOW), consistent with cardiac, renal, and liver enzyme activity. The 0.5 mg/kg EOW group showed the best efficacy, with liver and heart *α*-Gal A activity at 8 and 4 times that of the wild type, respectively, and the kidney maintained 25%. After 3 or 5 repeat doses, there was no increase in anti-α-Gal A antibodies, and enzyme activity accumulation was significantly better than with a single dose, with further accumulation of *α*-Gal A activity in the multi-dose group indicating no neutralizing anti-*α*-Gal A antibodies were produced (67). *In vitro* experiments showed that HEK293 cells transfected with α-Gal A mRNA had significantly higher mRNA and protein expression levels of α-Gal A and significantly increased enzyme activity in the cell lysate compared to the negative control; *in vivo* experiments, after mRNA-LNP injection in Fabry disease mice, α-Gal A activity in plasma significantly increased within 6 h and maintained a high level from 6 to 24 h, with significantly higher enzyme activity in the heart, liver, and kidneys than the control group. Immunohistochemistry showed that at 24 h, *α*-Gal A protein expression was enhanced in these tissues, and the effect was more significant in the high-dose group. At the same time, the levels of NF-κB, IL-6, and TNF-α in the liver of high-dose mRNA group mice were significantly reduced, and metabolomics identified 20 significantly different metabolites (VI*P* > 1, *p* < 0.05), with increased arachidonic acid, 5-HETE, and phosphatidylinositol 16/17/18, and decreased adenosine 5′-diphosphate, tryptophan, and glycolysis intermediates, mainly enriched in arachidonic acid, amino acids, and glycolysis/gluconeogenesis pathways; PCA/OPLS-DA showed dose-dependent metabolic profile separation, with significant changes in the high-dose group, suggesting that α-Gal A mRNA corrects the enzyme defect while reshaping the global metabolic homeostasis (66).

In terms of application, mRNA therapy can theoretically be used in various types of FD patients, particularly those with poor response to conventional ERT or the presence of anti-drug antibodies. Although mRNA therapeutics show promise in FD treatment, several practical challenges remain. These include optimizing the delivery system of mRNA, improving its targeting and stability, and determining the optimal dosage and frequency of administration.

## 6 AAV-mediated gene therapy

Viruses possess the natural ability to invade cells and can break through cellular barriers to deliver their genetic material into the cell interior, achieving stable gene expression in host cells. Among various viral vectors, the adeno-associated virus (AAV) vector is one of the preferred vectors in gene therapy due to its unique advantages (68). AAV is characterized by its small size, stable gene expression, low immunogenicity, and adjustable targeting, making it widely applicable in gene therapy. Wild-type AAV consists of single-stranded DNA that carries the *Rep* and *Cap* genes. The *Rep* gene encodes four proteins that control viral replication, packaging, and genome integration, while the *Cap* gene encodes the three major subunits that constitute the viral capsid: VP1, VP2, and VP3 (69). At the ends of the AAV genome, inverted terminal repeat sequences (ITRs) are present. These sequences are essential for the replication and packaging of the viral DNA (70).

**Figure 5:**
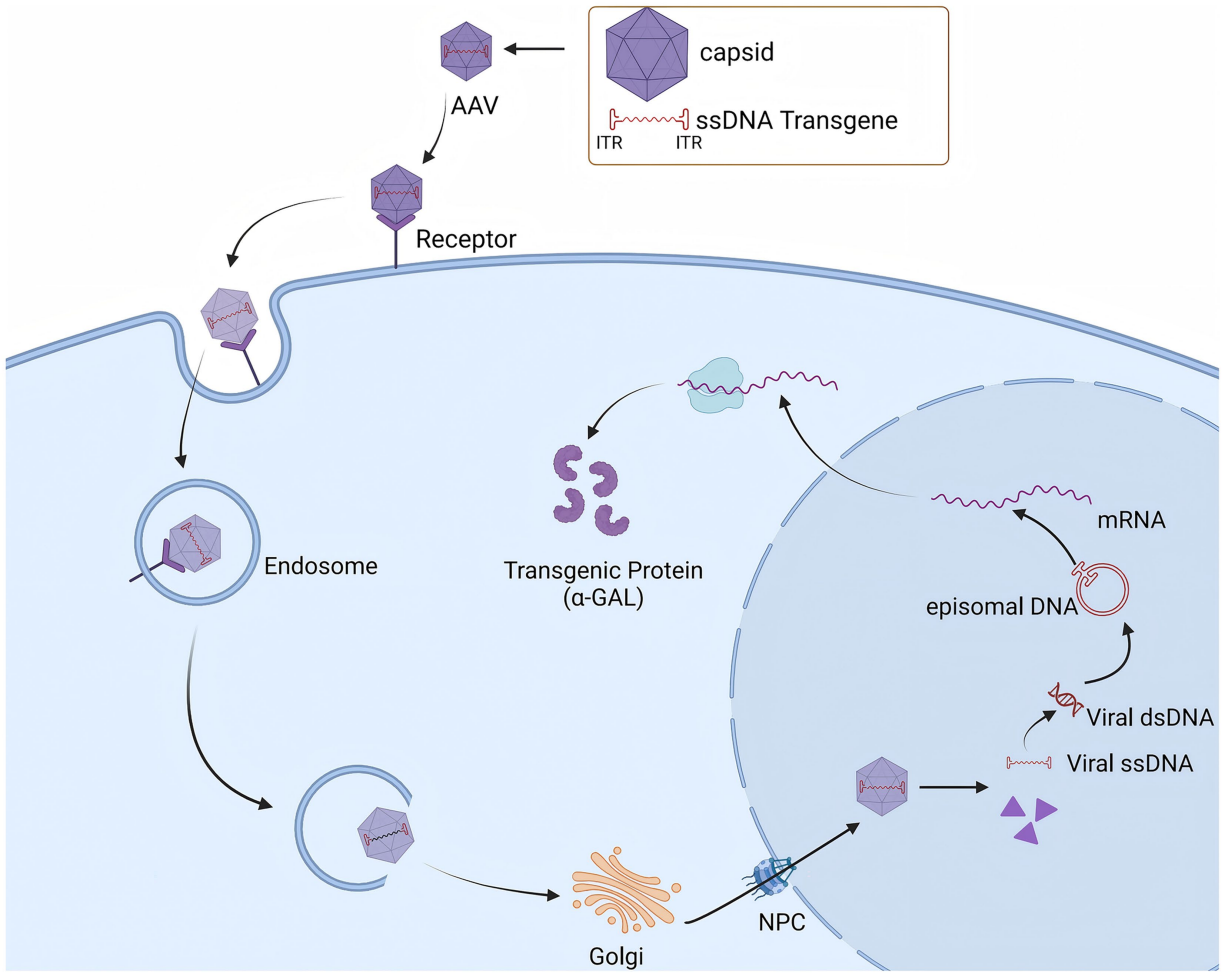
AAV vectors mediate specific target cell infection to facilitate *GLA* Gene delivery and α-GAL Expression.

Recombinant AAV (rAAV) is an AAV vector modified through genetic engineering technology. During the construction of rAAV, all gene sequences encoding the *Rep* and *Cap* proteins in the AAV genome are removed, retaining only the ITRs at both ends of the genome. These ITRs act as packaging signals to ensure that exogenous genes are correctly packaged into the viral particle (68, 71). With these modifications, rAAV vectors no longer contain the genes required for viral replication, thereby reducing the likelihood of replication in host cells and enhancing their safety. Upon entering the body, the rAAV vector is capable of specifically infecting target cells and delivering the therapeutic genes it carries into the cell interior (72). Within the cell, these therapeutic genes are expressed, and the corresponding proteins are produced, thereby compensating for proteins that are missing or functioning abnormally in the patient’s body due to genetic defects and exerting a therapeutic effect (Figure 5).

**Figure 6:**
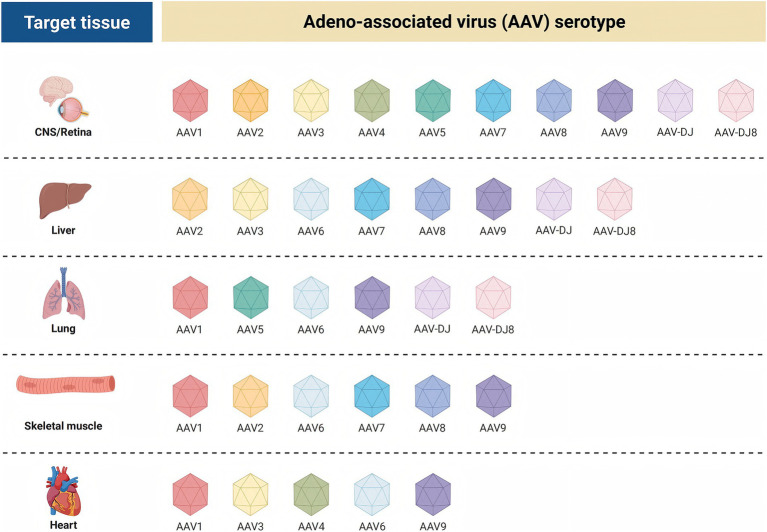
Tissue-specific affinities of adeno-associated virus AAV serotypes for different target tissue.

AAV has multiple serotypes, and the capsid of each serotype determines its affinity for different tissues and cells (Figure 6). These natural differences in tropism provide the basis for gene therapy targeting specific tissues or organs (72–74). Capsid engineering optimizes the properties of rAAV by modifying the rAAV capsid protein, offering a more effective tool for gene therapy and other biomedical applications. The modification strategies mainly include adjusting the tissue tropism and reducing immunogenicity, thereby enabling precise delivery of therapeutic genes to target cells. This approach significantly improves the utilization of therapeutic genes and therapeutic efficacy while reducing infection of non-target cells and tissues, and minimizing side effects caused by off-target effects (75, 76).

In mouse studies, the genome was pseudotyped with AAV8 for efficient transduction. AAV vectors exhibit favorable biosafety and tissue-targeting profiles, enabling precise delivery of the human *GLA* gene into liver cells. The liver-specific promoter FRE1 ensures that the human *GLA* gene is predominantly expressed in the liver, thereby avoiding non-specific expression in other tissues and enhancing therapeutic precision and safety. Pseudotyping with AAV8 further enhances the infectivity of the vector to target cells, enabling more liver cells to take up and express the human *GLA* gene (77).

ST-920 utilizes the AAV2/6 vector, which is capable of delivering the human *GLA* gene from the liver. This vector carries human *GLA* cDNA driven by a liver-specific expression cassette into patients with FD. The liver-specific expression cassette ensures that human *GLA* gene expression is initiated primarily in liver cells, thereby avoiding non-specific expression in other tissues and improving therapeutic precision. The human *GLA* gene that enters the nucleus of liver cells expresses functional *α*-Gal A under the action of intracellular transcriptional and translational mechanisms. In experiments using the Fabry mouse model, AAV2/6 vector-mediated gene therapy significantly increased α-Gal A activity in plasma and tissues, and the levels of Gb3 and Lyso-Gb3 in key pathological sites were essentially restored to normal. These findings indicate that α-Gal A successfully decomposed substrates, reducing their accumulation in the body, thereby alleviating damage to the kidneys, heart, and other organs caused by substrate accumulation and improving the pathological process of the disease (78).

While AAV-mediated gene therapy holds promise, immunogenicity and safety remain key considerations. Although rAAV typically does not integrate into the host genome, rare integration events can occur. These events are somewhat stochastic and may insert into key gene loci, thereby interfering with normal gene expression and regulation and potentially affecting the normal physiological functions of cells. Upon entering hepatocytes, AAV vectors may integrate their genomes into the host cell genome, thereby triggering hepatocyte injury (79). The vector dose is closely related to hepatotoxicity, with higher doses of AAV vectors being more likely to induce hepatotoxicity. When a large amount of vector enters the liver, it may exceed the processing capacity of hepatocytes, leading to metabolic overload and triggering cellular injury. In studies involving high-dose intravenous injection of AAV vectors in non-human primates and pigs, some animals exhibited marked hepatotoxicity, characterized by significant elevation of aminotransferases, hepatocellular damage, and even acute liver failure (80). The immune system’s recognition and response to AAV vectors are major contributors to hepatotoxicity. AAV vectors are recognized as foreign substances by the immune system, which triggers an immune response. Immune cells, such as macrophages and dendritic cells, recognize AAV vectors, activate relevant signaling pathways, and release pro-inflammatory cytokines, including TNF-*α* and IL-6. These cytokines induce an inflammatory response in the liver, leading to hepatocyte damage. The adaptive immune response is also involved. When T cells and B cells are activated, T cells attack AAV-infected hepatocytes. Antibodies produced by B cells may bind to antigens on the surface of AAV vectors or infected cells, forming immune complexes that are deposited in the liver and further exacerbate liver damage (81–83). Neurotoxic manifestations are also significant. Several studies have demonstrated that AAV vectors administered via different routes (e.g., intravenous, intrathecal injection, etc.) may lead to dorsal root ganglion toxicity, characterized by neuronal degeneration and necrosis, nerve fiber degeneration, and monocyte infiltration. In the central nervous system, motor dysfunction was observed in rats after intrathalamic injection of AAV-Cre. Similarly, in AAV-mediated gene therapy for FD mouse models, high-dose AAV injection was found to increase the expression of CXCL9 and CXCL10, recruit lymphocyte infiltration, and affect central nervous system function (84).

Theoretically, AAV therapy is expected to achieve the goal of “once-and-for-all, long-term benefit.” In a study involving 18 male patients with hemophilia A treated with an AAV vector (SPK-8011), factor VIII expression was maintained in 16 patients, with 12 followed for more than 2 years. No significant decrease in factor VIII activity was observed at weeks 26–52 and beyond, and the annualized bleeding rate was reduced by 91.5%. However, not all patients achieved such desirable outcomes, and some patients lost factor expression due to cellular immune responses against the AAV capsid. This suggests that there are individual differences in the durability of AAV therapy with immune responses being one of the key factors influencing durability (85). In a study for the treatment of MPS IIIA, healthy Beagles were administered an cerebrospinal fluid (CSF) AAV9 vector carrying the canine sulfonamidase gene. Results showed consistent detection of sulfonamidase activity in the CSF throughout the 7-year study period, with widespread sulfonamidase expression and activity in brain tissue, spinal cord, and dorsal root ganglia, and no obvious histologic abnormalities (86). In addition, the durability of AAV therapy is also closely related to vector design, disease progression, and other factors. For example, in the case of Leber’s congenital amaurosis (LCA) caused by RPE65 deficiency, early studies using AAV vector therapy improved visual function in some patients, but the duration of efficacy was limited by the progression of retinal degeneration. However, through continuous optimization of vector design, such as the development of new AAV2/5-OPTIRPE65 vectors, stronger effects than those of the original vectors were demonstrated in mouse experiments (87).

In terms of vector immunogenicity, preclinical studies consistently show that humoral responses are mild and do not affect efficacy. Chang et al. used *GLA*−/y mice as a model, administering a single moderate dose (1 × 10^11^ vg/mouse) of AAV8-*GLA* or AAV9-*GLA* via the tail vein, and monitoring serum anti-*GLA*-IgG titers at baseline and weeks 2, 4, 6, and 8 after administration: all were below the threshold (mean + 3 SD) during the first 4 weeks, with only a slight increase at week 8, indicating an extremely limited humoral immune response to the transgene (88). Takeda’s PET tracing study further confirmed that [18F]AGAL heart signals persisted for 26 weeks and were highly correlated with *in vitro* enzyme levels (*r* > 0.9), indicating that anti-*GLA* antibodies did not significantly neutralize the expressed enzyme (89). Additionally, using the enzyme-stabilized mutant Eng-C, the vector dose can be reduced to ≤5 × 10^11^ vg/kg while maintaining equivalent efficacy, further lowering the capsid protein burden and potential immune activation risk (90).

## 7 Symptomatic treatments

### 7.1 Protective role of ACEIs/ARBs in FD kidneys

Angiotensin-converting enzyme inhibitors (ACEIs) reduce the production of angiotensin II by inhibiting angiotensin-converting enzyme. Angiotensin receptor blockers (ARBs) selectively block the binding of angiotensin II to receptor 1 (AT1), effectively inhibiting its vasoconstrictor effect. This action reduces the elevated capillary pressure in the glomeruli and lowers the intraglomerular pressure. This makes it more difficult for proteins to pass through the glomerular filtration membrane into the urine when blood flows through the glomeruli, thereby reducing urine protein levels (91). In a prospective observational study of 24 adult FD patients undergoing ERT, the effect of anti-proteinuria therapy with ACEIs/ARBs on renal function was evaluated, and the results showed that 75% of patients achieved the urine protein-to-creatinine ratio (UPCR) target with ACEIs/ARBs therapy, and ACEIs/ARBs treatment reduced proteinuria and slowed the decline in eGFR even when baseline eGFR was low. Although some patients have decreased renal function due to severe baseline renal impairment, ACEIs/ARBs remain a key means of controlling proteinuria and delaying the progression of kidney disease (92). The overall therapeutic goal of ACEIs/ARBs in combination with ERT is to reduce urinary protein excretion to less than 500 mg/day and stabilize the decline in renal function to −1 mL/min/1.73 m2/year. In the absence of ACEIs/ARBs, ERT alone does not reduce proteinuria in Fabry patients (93). With the increasing clinical use of sodium-glucose cotransporter-2 (SGLT2) inhibitors and mineralocorticoid receptor antagonists (MRAs), these drugs may confer renal benefits in FD patients.

### 7.2 Cardiovascular system

A long-term follow-up study of adult FD patients found no significant differences in cardiac symptoms and arrhythmias in patients treated with ERT compared with untreated patients (19). This may be due to the fact that the mechanism of arrhythmogenesis is more complex and is associated with abnormal ion channel function, inflammatory response, and fibrosis, in addition to metabolic substrate accumulation (94, 95). Anticoagulants, such as warfarin and rivaroxaban, are among the important therapeutic agents that can effectively reduce the risk of thromboembolic events (96). *β*-blockers can be used to control the ventricular rate in patients and alleviate symptoms such as palpitations. Antiarrhythmic drugs, such as amiodarone, can also be used to restore and maintain sinus rhythm (97). However, their use may affect other bodily functions, such as thyroid function, lysosomal pH and enzyme activity, and thus requires close monitoring in patients with FD. Catheter ablation, including radiofrequency ablation, is an effective therapeutic option that restores normal cardiac rhythm by disrupting abnormal electrical conduction pathways. For some patients with severe disease who do not respond well to medications, it may be necessary to consider implanting a pacemaker or implantable cardioverter defibrillator (ICD). Implanted pacemakers can be used to treat severe bradycardia or heart block, while ICD can prevent sudden death by administering a shock defibrillator in the event of a life-threatening arrhythmia (94, 98, 99).

### 7.3 Neuralgia

In actual treatment, some patients continue to experience neuropathic pain symptoms despite overall improvement in their condition with ERT. Antiepileptic drugs, such as gabapentin and carbamazepine, relieve neuralgia by modulating neuronal excitability and reducing nerve impulse transmission, and are widely regarded as first-line drugs for treating neuropathic pain in FD (100). A study showed that male FD patients aged 15–45 years treated with gabapentin for 4 weeks experienced a reduction in pain compared to pre-treatment levels, and the treatment was generally well-tolerated (101). Additionally, a case report documented a 7-year-old patient experiencing intermittent, intense “burning” sensations in the hands and feet, which were unresponsive to aspirin, acetaminophen, acetylcodeine, and phenytoin. Carbamazepine and phenytoin sodium reduced the frequency and duration of pain episodes to 3–4 per year, indicating that while antiepileptic drugs significantly decreased the frequency of FD-related neuralgia, they did not provide complete relief. Over 28 consecutive months of observation, 7 crises occurred. During these crises, intravenous morphine was administered at 0.06 mg/kg, followed by a continuous infusion of 0.02 mg/kg/h of the drug and 0.25 mg/kg of amitriptyline at bedtime, which rapidly alleviated the pain symptoms (102). A recent study analyzing the surface proteome of sensory-like neurons derived from induced pluripotent stem cells (IPSCs) in FD patients identified 48 abnormally expressed surface proteins, among which pain-related proteins like CACNA2D3 and GPM6A were significantly dysregulated. Surface protein interaction network analysis revealed enrichment of extracellular matrix proteins (e.g., COL5A1, POSTN) and transmembrane receptors (e.g., ROBO2, SEMA6D), suggesting impaired neuronal network integrity and axon guidance abnormalities. Although there were no significant differences in mRNA levels of related genes, the protein interaction network unveiled potential abnormalities in neuronal plasticity and inflammatory pathways. Combining the core lysosomal dysfunction in FD, it is hypothesized that Gb3 accumulation indirectly affects ion channels and the neurochemical environment through membrane structural changes and impaired secretion, collectively contributing to the development of neuropathic pain (103).

## 8 Accessibility and economic feasibility of Fabry disease treatment drugs

Orphan drugs for the treatment of FD exhibit a significant “north–south divide” in terms of accessibility and affordability worldwide: High-income countries rely on institutional collaboration and policy innovation to significantly optimize drug access and health insurance coverage, continuously improving patient accessibility; while middle and low-income countries are trapped in a “high clinical value—low actual accessibility” dilemma due to structural barriers such as delayed approval, absent health insurance, and excessive concentration of medical resources. At the same time, although orphan drugs for FD have clear efficacy, they exert a sustained squeeze on health insurance funds due to high pricing and an imbalanced cost-effectiveness ratio, further amplifying global health inequalities.

### 8.1 Accessibility disparities: well-developed systems in high-income countries vs. multiple barriers in middle and low-income countries

In high-income countries, the approval, supply, and use system for orphan drugs has become mature. In the approval process, the United States, through the Orphan Drug Act, provides 7 years of market exclusivity, 50% tax credit for clinical research and development, free designation, and full technical support, significantly speeding up the time to market; the EU, based on Regulation (EC) No 141/2000, grants 10 years of market exclusivity and fee reductions, and through the “centralized approval” and “mutual recognition” mechanisms, allows drugs to be marketed simultaneously or rapidly sequentially in member states, greatly simplifying the process (104–106). In the payment process, Germany’s statutory health insurance fully reimburses almost all OMPs approved by the EMA, despite the annual drug cost for Fabry disease reaching 264,896 euros (107), the patients’ actual burden is extremely low. In the use process, Germany, Norway, Italy, and other countries incorporate home infusion into routine management (107–109), making home treatment more comfortable, less stressful, and more effective, significantly improving the accessibility of treatment and patients’ compliance.

Compared to high and middle-income countries, there are significant barriers to the accessibility of orphan drugs for Fabry disease in low-income countries. A study in India shows that only 51.8% of 54 patients could receive enzyme replacement therapy, with an average diagnosis delay of 11.7 years (110). Although Turkey has included 43 orphan drugs in its reimbursement list, 22 of them have not been launched domestically and require individual applications for import by the social security agency (111). Among the 242 new orphan drugs approved by the US FDA from 2013 to 2023, as of January 1, 2025, China has only approved 119, with a significant delay: the median lag time for approved varieties is 1,004 days, with the longest delay reaching 3,785 days, and there is still a significant lag compared to Asian countries such as Japan and South Korea. Before being included in the national health insurance in a first-tier city in Northeast China, 15 out of 16 FD orphan drugs exceeded the “catastrophic medical expenditure” threshold for rural residents; even after inclusion in the health insurance, 8 of them still did not meet the standard (112). The National Essential Medicines List of Thailand only includes 22.93% of registered orphan drugs, and five-year procurement data show that only 31.7% of registered varieties were actually supplied to hospitals (113), reflecting the triple accessibility barriers of “diagnosis delay—market lag—reimbursement gap.”

### 8.2 Economic differences: high drug costs dominate vs. healthcare burden and cost-effectiveness imbalance

Globally, the payment for orphan drugs for FD continues to place a heavy burden on public finances and healthcare systems. Canada once tried a “triple-sharing” model: pharmaceutical companies invested in the University Health Network (UHN) through provincial governments to support Canadian Fabry Disease Initiative (CFDI) research; provincial governments procured drugs with assistance from the federal government, creating a hybrid mechanism where “companies bear the research costs and governments share the procurement costs” to spread the risk of a single entity. However, after about a year, the federal government exited, citing “a lack of a clear endpoint.” The core economic consideration was that the ERT involved in the CFDI was one of the most expensive treatment methods globally at the time, requiring continuous funding to maintain patient registration systems, collect real-world evidence, and with no clear end to the research period. The federal government may have been concerned that such long-term, indefinite funding would crowd out resources in other public health areas, and without a national budget framework for rare disease treatment, it would be difficult to ensure sustained financial support, so it chose to withdraw to avoid long-term financial risks (114). The case in Chengdu, China further highlights the impact of high-priced orphan drugs on local finances. In 2019, the total budget for 98 rare disease patients’ orphan drugs, if fully self-paid, reached 1.79 billion yuan; under six simulated reimbursement scenarios, it still required 0.32–1.56 billion yuan, and the annual funding needs are expected to rise to 2.00–13.03 billion yuan over the next three years (115). India, as a traditional populous country, insufficient coverage exacerbates fiscal fragility. Only 37.2% of the population has any form of health insurance, with significant inter-state and population differences; the National Health Account (NHA) data shows that while government health spending has increased year by year, the proportion paid by individuals remains high, and high-priced orphan drugs are prone to push families into catastrophic spending (115). It is evident that whether in high-income or middle- and low-income countries, how to establish a sustainable financing and risk-sharing mechanism for rare drugs within limited budgets has become a global fiscal governance challenge.

In the cost-effectiveness dimension, although ERT can bring measurable clinical benefits, it is accompanied by the sharp contradiction of “high gain, higher price” in the long term. Agalsidase beta can delay renal function decline, but the incremental health benefits are extremely limited: the Dutch cohort study shows that lifetime Quality-adjusted life years (QALYs) increase by only 1.6 years, and the years free of end-organ damage (YFEOD) are extended by 1.5 years, with slightly higher benefits for males. The corresponding Incremental cost effectiveness ratio (ICER) reaches 6.1 million euros/QALY, the incremental cost of YFEOD is 6.6 million euros/year, about 8 times that of Gaucher’s ERT at the same time; sensitivity analysis suggests that even if the drug price is reduced by 25% (from 200,000 to 150,000 euros/year), the ICER is still 4.55 million euros/QALY, far exceeding the Dutch healthcare threshold (≈50,000 euros/QALY). Drug costs account for 70–90% of the total treatment cost and are the only key variable determining the ICER (116). China reduced the ERT price to the lowest global range through national medical insurance negotiations in 2021, with an ICER of 148,072 yuan/QALY, within the 1–3 times per capita GDP threshold (89,358–268,074 yuan/QALY), theoretically making it economically viable (117). However, this ICER is still based on the “medical insurance payment price,” and if local fiscal support is insufficient, residents in economically underdeveloped areas need to bear out-of-pocket costs as well as hidden costs such as transportation and care, which still limit actual accessibility. Therefore, while maintaining the national negotiation price, it is necessary to increase the central-provincial fiscal subsidy ratio and establish a special co-payment fund for rare diseases to truly transform “cost-effectiveness acceptable” into “budget-burden acceptable” actual accessibility.

## 9 Conclusion

Significant advancements have been achieved in the pathogenesis, diagnosis, and treatment of FD as research progresses. However, numerous challenges persist. ERT, while effective in alleviating symptoms and delaying disease progression, is not without limitations. For patients with advanced organ damage, ERT provides only limited improvement and cannot reverse severe organ disease. Furthermore, due to the difficulty of ERT in penetrating the blood–brain barrier, it is challenging to improve central nervous system symptoms. Migalastat, a pharmacological chaperone therapy, is only effective for patients with specific missense mutations. Although it can stabilize kidney function and improve heart structure, it requires close monitoring due to the differences in efficacy between *in vivo* and *in vitro* effects. Emerging therapies, such as substrate reduction therapy, mRNA therapy, and AAV-mediated gene therapy, have demonstrated potential in the treatment of FD. Among these, AAV-mediated gene therapy, which leverages precise targeting and efficient expression through multiple serotypes of vectors, has significantly ameliorated the pathological process of FD in animal experiments and is theorized to provide long-term benefits to patients. However, it faces safety concerns, including the optimization of dosing regimens, enhancement of delivery systems, and management of immunogenicity (Table 3).

**Table 3:** Comparison of the pharmacological mechanisms, organ benefits, and limitations of the main treatment strategies for Fabry disease.

Therapeutic therapies	Pharmacological mechanism	Cardiovascular system	Kidney	Nervous system	Advantage	Limitations
Enzyme replacement therapy	Intravenous administration of recombinant α-Gal A promotes the degradation of Gb3 and Lyso-Gb3, reducing substrate accumulation in tissues	1. Long-term ERT treatment can maintain LVMI stability, even slight decline, and keep the annual change slope of PWTd within the normal range. 2. But ERT still cannot completely eliminate arrhythmias or microvascular risks, and MACE such as sudden cardiac death may still occur.	1. ERT can significantly reduce Gb3 deposition in the renal mesangial area and increase creatinine clearance; 2. Early initiation of ERT can more significantly slow the decline in renal function;	ERT can significantly alleviate neuropathic pain, reduce pain scores and the frequency and severity of pain attacks; and improve the threshold for cold and hot sensations in hands and feet, enhance sweating function and heat tolerance; however, it cannot fully restore the function of the peripheral nervous system	ERT clinical applications are mature, with 3 approved drugs available for selection; it can significantly delay the progression of multi-organ disease and improve long-term patient prognosis	1. The burden of ERT treatment affects patient compliance; 2. ERT has immunogenicity, and some treatment regimens have a high antibody positivity rate, which can neutralize enzyme activity; 3. α-Gal A has difficulty penetrating the blood–brain barrier and provides limited improvement for central nervous system symptoms;
Pharmacological chaperone therapy	Migalastat can specifically and reversibly bind to the active site of the mutated α-Gal A produced by GLA gene mutations, stabilize the three-dimensional structure of the enzyme, and enhance the stability and activity of the mutated enzyme	Compared to ERT, patients treated with Migalastat showed a more significant decrease in LVMI	Migalastat can significantly reduce Gb3 deposition in the renal glomerular mesangial area and can stabilize creatinine clearance	Migalastat can improve symptoms related to autonomic nerve function and has a certain relieving effect on neuropathic pain;	1. Oral administration offers high convenience and excellent patient compliance; 2. Migalastat has a good safety profile, serious adverse events are rare in long-term treatment, and the tolerability of adolescent patients is consistent with that of adults; 3. Patients with end-stage renal disease do not need to adjust the dose, and drug removal during dialysis does not affect the efficacy.	1. Migalastat is only effective for specific missense mutations. 2. The therapeutic potential in vitro may not be fully consistent with in vivo efficacy; for some “combinable mutation” patients, the therapeutic effect is still inferior to ERT.
Substrate reduction therapy	Venglustat is a GCS inhibitor that reduces the synthesis of Gb3 by inhibiting enzymatic reactions	Venglustat can significantly reduce myocardial Gb3 and lyso-Gb-3 deposition, but there is limited direct clinical efficacy data related to the heart	Venglustat can significantly reduce renal Gb3 and lyso-Gb-3 deposition, but data on its direct clinical efficacy in relation to the kidneys are limited;	Venglustat can cross the blood–brain barrier, reduce the accumulation of glycosphingolipids in the brain, and compensate for the defect of ERT not being able to act on the central nervous system	Venglustat can be administered orally, with good patient compliance; it can also cross the blood–brain barrier, improving symptoms of central nervous system involvement	Currently, only phase IIa and expanded clinical trials have been conducted, lacking large-scale phase III clinical data to validate efficacy and safety
mRNA therapy	Through intravenous injection of mRNA that expresses α-Gal A, it promotes an increase in the expression of functional α-Gal A in cells within the body, reducing the accumulation of Gb3 and Lyso-Gb3	After multiple-dose administration, α-Gal A activity in cardiac tissue significantly increased, and the deposition of Gb3 in cardiomyocytes decreased.	After mRNA injection, significant reduction in renal Gb3 deposition, stable eGFR, no significant change in creatinine, and no renal histopathological damage	mRNA theoretically can reduce Gb3 deposition in nervous tissue and improve neurological symptoms by expressing α-Gal A, but currently it is mainly preclinical data and lacks clinical efficacy data for the nervous system	mRNA can rapidly increase α-Gal A levels in the body, and its efficacy does not diminish with repeated administration; it is also widely applicable and theoretically usable in patients who do not respond well to ERT or have antibodies against it; it also has good safety, with only a few doses in non-human primates causing transient mild infusion reactions, without complement activation or acute cytokine storms	mRNA therapy remains in the preclinical stage, lacking clinical trial data in humans; it also needs to optimize delivery systems, improve tissue targeting, and enhance mRNA stability
AAV-mediated gene therapy	By delivering the human GLA gene to target cells via rAAV vectors, tissue-specific expression is achieved using the virus-specific tissue tropism, producing functional α-Gal A to compensate for the enzyme defect	1. The AAV2/6 vector can restore normal levels of cardiac Gb3 and Lyso-Gb3 in Fabry mouse models, alleviating cardiomyocyte damage; 2. AAV8 vector drives GLA expression in mice via the liver-specific promoter FRE1, resulting in a significant increase in α-Gal A activity in heart tissue without the development of new cardiac fibrosis.	AAV2/6 vector in mouse models, renal Gb3 and Lyso-Gb3 levels return to normal, and significant reduction in glomerular endothelial, mesangial cell, and podocyte substrate deposition	1. The AAV9 vector can be administered via cerebrospinal fluid, achieving widespread enzyme expression in the brain, spinal cord, and dorsal root ganglia in canine models, providing a possible avenue for improving central nervous system symptoms. 2. However, high doses of AAV may increase CXCL9/CXCL10 expression, recruit lymphocytes infiltration, and affect central nervous function.	1. Theoretically, AAV-mediated gene therapy can achieve long-term benefits. 2. Tissue targeting and expression efficiency can be improved through serotype optimization and promoter regulation; 3. Some vectors can act on the central nervous system, making up for the shortcomings of ERT and chaperone therapy.	1. AAV-mediated gene therapy has immunogenicity and toxicity risks 2. Although the risk of vector integration is low but still exists, rare integration may interfere with host gene expression; 3. Data from human clinical trials are limited, and long-term efficacy and safety need further verification.

The future of FD treatment will be centered on the goal of “precision, efficiency, and cure.” This will be achieved through the optimization of existing therapies, breakthroughs in emerging technologies such as mRNA targeted delivery, AAV capsid modification, and CRISPR gene editing, reliance on precision medicine for early screening and subtyping, and enhancement of patient care quality through interdisciplinary integration and long-term management. Interdisciplinary integration and long-term management will improve the quality of patients’ full-cycle care. With the synergy of policy support and technological innovation, FD is expected to become a model for gene therapy in rare diseases, facilitating the transition from “lifelong replacement therapy” to “gene-level cure.”

## Glossary

FD: Fabry Disease
LSD: Lysosomal storage disease
*α*-GAL: α-galactosidase A
Gb3: Globotriosylceramide
Lyso-Gb3: Globotriosylsphingosine
ERT: Enzyme replacement therapy
LVH: Left ventricular hypertrophy
Ccr: Creatinine clearance
eGFR: Estimated glomerular filtration rate
RCT: Randomized controlled trial
GCS: Glucose ceramide synthase
SSCE: Superficial skin capillary endothelium
AAV: Adeno-associated virus
ITRs: Inverted terminal repeat sequences
rAAV: Recombinant AAV
CNS: Central nervous system
CSF: Cerebrospinal fluid
LCA: Leber’s congenital amaurosis
ACEI: Angiotensin-converting enzyme inhibitor
ARB: Angiotensin receptor blocker
AT1: Angiotensin II to receptor 1
SGLT2: Sodium-glucose cotransporter-2
MRA: Mineralocorticoid receptor antagonist
ICD: Implantable cardioverter defibrillator
IPSC: Induced pluripotent stem cell
UHN: University Health Network
CFDI: Canadian Fabry Disease Initiative
NHA: National Health Account
QALY: Quality-adjusted life year
YFEOD: years free of end-organ damage
ICER: Incremental cost effectiveness ratio
